# Supplementary material for: Role of EXO1 nuclease activity in genome maintenance, the immune response and tumor suppression in Exo1D173A mice
Source: Nucleic Acids Res. 2022 Jul 18;50(14):8093–106. doi: 10.1093/nar/gkac616 (PMC9371890; doi:10.1093/nar/gkac616)
Supplement: gkac616_Supplemental_File [file gkac616_supplemental_file.pdf]

Figure S1

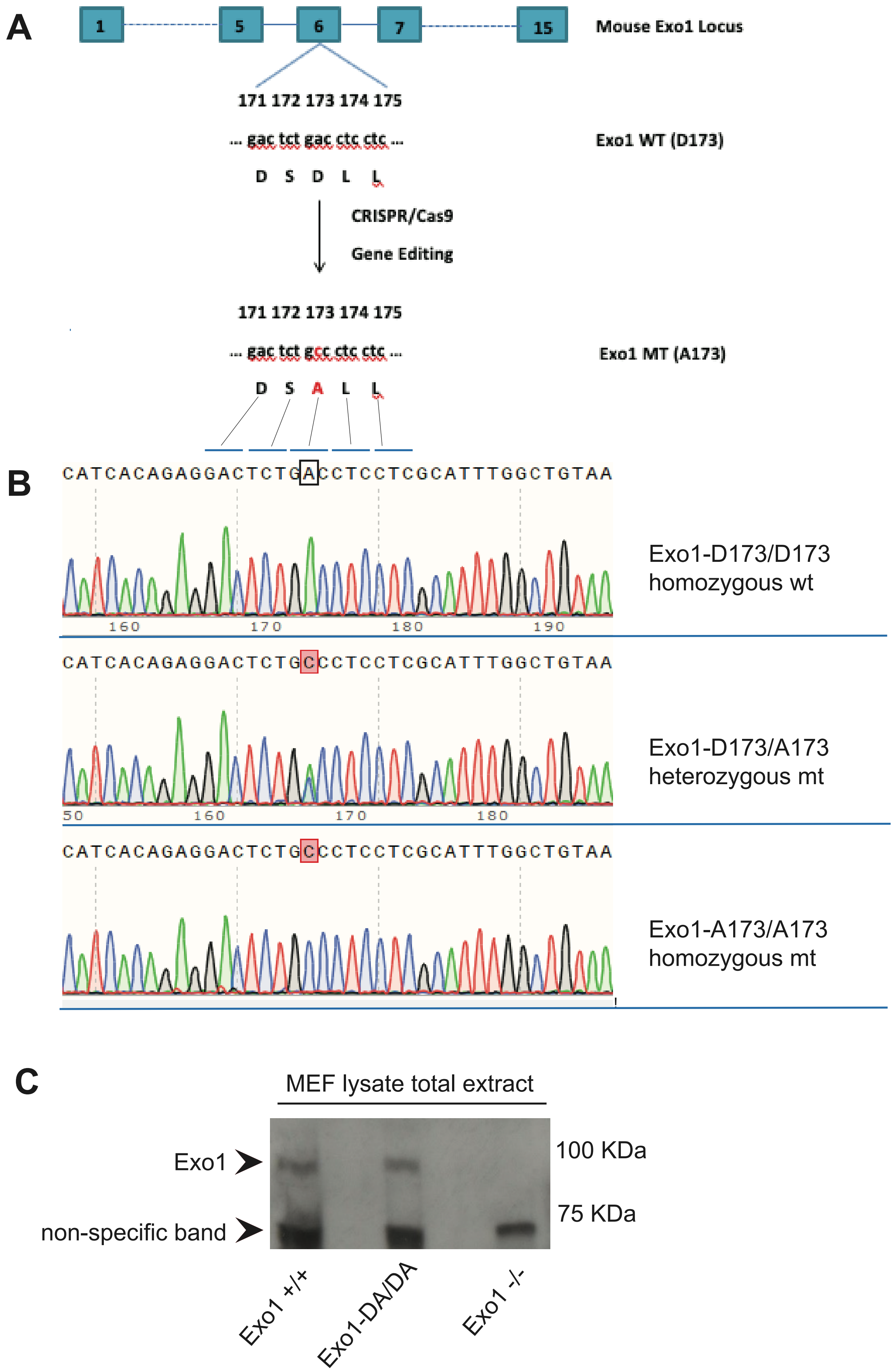

Figure S1: Generation of *Exo1*$^{DA/DA}$ mice. A) The mouse Exon1 DA allele was generated by CRISPR/Cas9 mediated gene editing and carries a one-nucleotide substitution mutation (A to C) at codon 173 in exon 6 of the Exon1 gene to generate the amino acid substitution D173A. B) Mouse cohorts were screened using PCR and Sanger sequencing to identify offspring carrying the desired genetic mutation, as depicted. C) Further western blot analysis using mouse embryonic fibroblasts extracts from various cohorts along with an anti-Exo1 antibody confirms the maintenance and stability of Exo1 protein in wild type and DA mutant cells.

Figure S2

A.

	ΔΔG stability (kcal/mol)
DynaMut2[1]	1.18
DynaMut[2]	0.671
DeepDDG[3]	-1.092
DUET[4]	0.595
SDM[5]	-0.81
PremPS[6]	0.03
mCSM [7]	0.009

B.

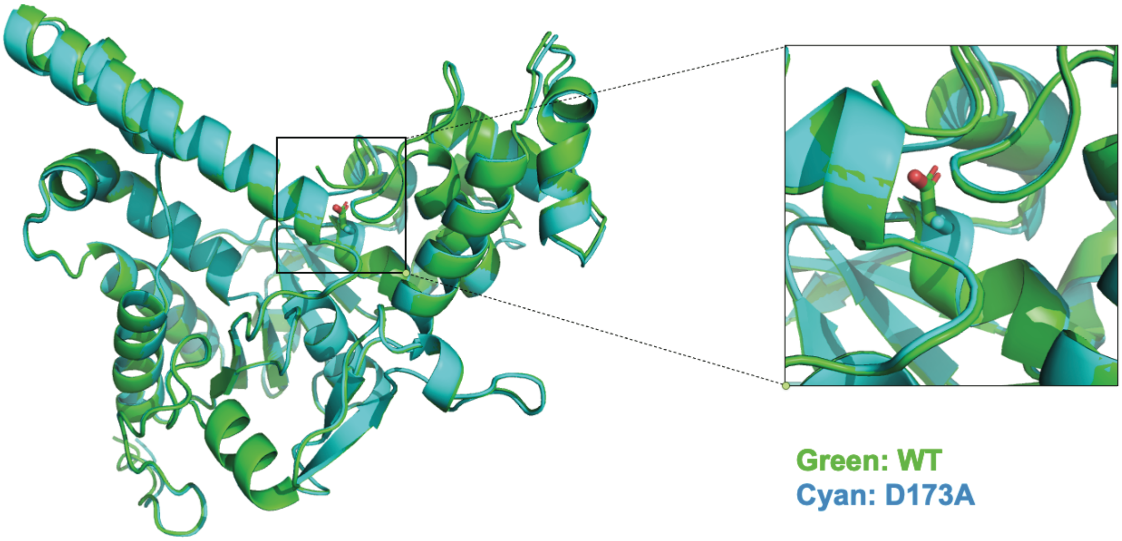

Figure S2: Structural analysis of EXO1-D17A protein compared to EXO1-WT. A) Structure based stability prediction upon D173A mutation. PDB file 3qeb was used as the WT structure, and ΔΔG was calculated using web based programs. Positive values mean increased stability, while negative values suggest decreased stability upon mutation. Generally, the programs do not predict a large change in ΔΔG, suggesting a minor or no change in structural perturbation upon mutation. B) Structural comparison of the nuclease domains of WT and D173A. The structures of the nuclease domains were calculated using AlphaFold using amino acid 1-352. Residues beyond residue 352, predicted to be highly unordered without a stable structure, were not used in the structural prediction. The two structures have an RSMD of 0.3 Å, suggesting D173A mutation does not bring in significant structural changes.

References

1. Rodrigues, C. H. M.; Pires, D. E. V.; Ascher, D. B., DynaMut2: Assessing changes in stability and flexibility upon single and multiple point missense mutations. *Protein Science* **2021,** *30* (1), 60-69.
2. Rodrigues, C. H.; Pires, D. E.; Ascher, D. B., DynaMut: predicting the impact of mutations on protein conformation, flexibility and stability. *Nucleic Acids Research* **2018,** *46* (W1), W350-W355.
3. Cao, H.; Wang, J.; He, L.; Qi, Y.; Zhang, J. Z., DeepDDG: Predicting the Stability Change of Protein Point Mutations Using Neural Networks. *J Chem Inf Model* **2019,** *59* (4), 1508-1514.
4. Pires, D. E.; Ascher, D. B.; Blundell, T. L., DUET: a server for predicting effects of mutations on protein stability using an integrated computational approach. *Nucleic Acids Res* **2014,** *42* (Web Server issue), W314-9.
5. Worth, C. L.; Preissner, R.; Blundell, T. L., SDM--a server for predicting effects of mutations on protein stability and malfunction. *Nucleic Acids Res* **2011,** *39* (Web Server issue), W215-22.
6. Chen, Y.; Lu, H.; Zhang, N.; Zhu, Z.; Wang, S.; Li, M., PremPS: Predicting the impact of missense mutations on protein stability. *PLoS Comput Biol* **2020,** *16* (12), e1008543.
7. Pires, D. E.; Ascher, D. B.; Blundell, T. L., mCSM: predicting the effects of mutations in proteins using graph-based signatures. *Bioinformatics* **2014,** *30* (3), 335-42.
